# Transcriptional Silencing of MCL-1 Through Cyclin-Dependent Kinase Inhibition in Acute Myeloid Leukemia

**DOI:** 10.3389/fonc.2019.01205

**Published:** 2019-12-12

**Authors:** Raoul Tibes, James M. Bogenberger

**Affiliations:** ^1^NYU School of Medicine & Perlmutter Cancer Center, NYU Langone Health, New York, NY, United States; ^2^Mayo Clinic, Scottsdale, AZ, United States

**Keywords:** acute myeloid leukemia, BCL-2, CDK, CDK9, MCL-1, transcriptional inhibition

## Abstract

Acute myeloid leukemia (AML) is the most common adult acute leukemia. Survival remains poor, despite decades of scientific advances. Cytotoxic induction chemotherapy regimens are standard-of-care for most patients. Many investigations have highlighted the genomic heterogeneity of AML, and several new targeted therapeutic options have recently been approved. Additional novel therapies are showing promising clinical results and may rapidly transform the therapeutic landscape of AML. Despite the emerging clinical success of B-cell lymphoma (BCL)-2 targeting in AML and a large body of preclinical data supporting myeloid leukemia cell (MCL)-1 as an attractive therapeutic target for AML, MCL-1 targeting remains relatively unexplored, although novel MCL-1 inhibitors are under clinical investigation. Inhibitors of cyclin-dependent kinases (CDKs) involved in the regulation of transcription, CDK9 in particular, are being investigated in AML as a strategy to target MCL-1 indirectly. In this article, we review the basis for CDK inhibition in oncology with a focus on relevant preclinical mechanism-of-action studies of CDK9 inhibitors in the context of their therapeutic potential specifically in AML.

## Introduction

In 2018, an estimated 21,380 people in the United States were diagnosed with acute myeloid leukemia (AML), making AML the most common adult acute leukemia (1). AML accounts for up to 32% of all adult leukemias and 1.3% of all new cancer cases in the United States (1). Survival outcomes for patients diagnosed with AML are poor. For patients <60 years of age treated with conventional chemotherapy, 35–45% achieve long-term survival (2). Older patients (≥65 years of age) have an even worse prognosis, with long-term survival rates of only 10–15% (2).

Cytotoxic induction therapy (7 days of standard-dose cytarabine and 3 days of an anthracycline or an anthracenedione, e.g., daunorubicin or idarubicin; also known as 7 + 3), which was developed in the 1970s, remains the standard of care for most patients with AML undergoing induction treatment (3, 4); however, recently a novel liposomal formulation of daunorubicin and cytarabine was approved by the US Food and Drug Administration (FDA) for high-risk and secondary AML (5, 6). Hypomethylating agents (HMAs), such as azacitidine and decitabine, while not formally approved by the FDA for the treatment of AML as single agents, have been used as *de facto* standard-of-care therapy for elderly patients with AML unfit for induction therapy. Response rates for azacitidine and decitabine monotherapy are low in elderly patients (10–50%), with a median overall survival of <1 year (7). Even for patients who achieve a complete remission with standard therapy, most will ultimately relapse and face a poor prognosis (8). Thus, there is a clear need for improved therapeutic options in AML. Since 2013, 65 drugs have been granted orphan designation specifically for the treatment of AML (9); however, there were only four US FDA approvals for new treatments in AML in 2017 (6, 10–16) and another four in 2018 (17–26).

Venetoclax combination therapy in particular has yielded promising results in elderly patients, with recent clinical trials showing a 65% complete remission rate in patients ≥75 years (7). As venetoclax is a selective inhibitor of B-cell lymphoma 2 (BCL-2), these outcomes highlight the importance of targeting BCL-2 family proteins for the treatment of AML. However, resistance to BCL-2 inhibition on venetoclax combination regimens is emerging, and alternative strategies to address resistance mechanisms are needed. Indeed, increased advances in the understanding of the role of BCL-2 family proteins and their interactors in apoptosis and AML pathogenesis have led to the discovery and clinical development of additional investigational treatments. Recent functional screens using CRISPR/Cas9 approaches highlight the central importance of mitochondrial function/architecture in resistance to BCL-2 inhibitor venetoclax (27, 28). Other BCL-2 family protein members may also play a role in AML patients refractory/resistant to BCL-2 inhibition, particularly MCL-1, which is an antiapoptotic multidomain protein regulated by distinct cyclin-dependent kinases (CDKs) in both apoptotic and cell-cycling pathways (8, 29–31).

In this review, we will address advances in the clinical development of CDK inhibitors as a strategy for indirectly targeting MCL-1 in the treatment of AML. We will briefly discuss the BCL-2 family of proteins that underlie AML pathogenesis and treatment resistance, as well as the therapeutic potential of targeting CDKs that regulate transcription, focusing on CDK9 inhibition.

## BCL-2 Family Of Proteins, Including MCL-1, In AML Pathogenesis

**Figure 1:**
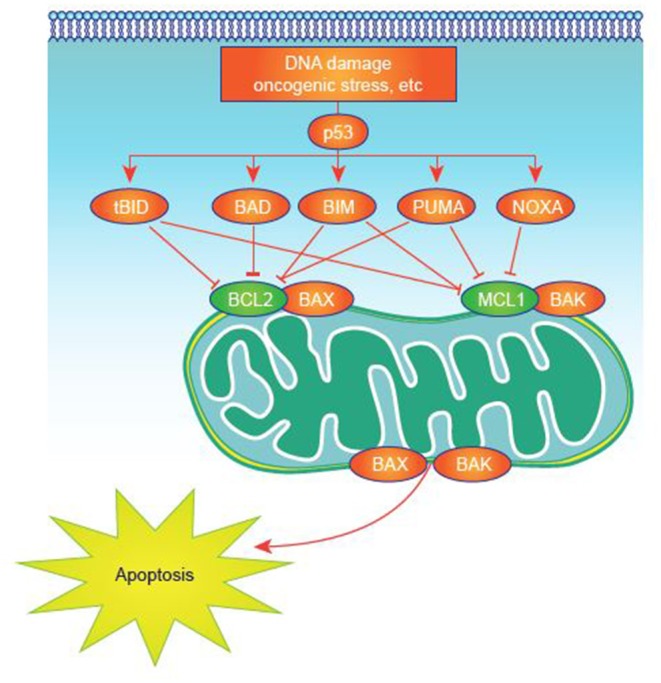
Apoptosis activation in normal and tumor cells. Apoptosis signaling is normally triggered by multiple death signals. There is a finely tuned balance between proapoptotic and antiapoptotic proteins that results in efficient apoptosis induction. MCL-1 and other antiapoptotic proteins block apoptotic effectors like BAK on the surface of the mitochondria. BH3-only proteins, such as NOXA, untether BAK from MCL-1, permitting BAK to cause events that result in cell death.

Many studies have sought to identify critical, pathogenic mechanisms in AML. However, these efforts are complicated by the inherent heterogeneity of the disease (32) and its relatively low mutational load compared with some malignancies (33). Deregulated expression of one or more of the apoptosis-controlling BCL-2 family members, central regulators of cell survival and apoptosis, is common in AML (34). This family of proteins, which includes more than 20 members, has pro- or antiapoptotic functions converging on mitochondrial apoptosis, also commonly known as intrinsic apoptosis (35, 36), a critical cell-death regulatory mechanism (Figure 1). Impairment of apoptosis represents one of the postulated hallmarks of cancer and is highly relevant to AML, as antiapoptotic mechanisms are upregulated in AML (35, 37, 38).

Based on sequence and structural homologies, BCL-2 family proteins can be classified into three groups, each containing at least one BCL-2 homology (BH) domain (BH1-4) (31):

- Proapoptotic multidomain effector proteins (including BAK, BAX, and BOK), which mediate the release of critical proapoptotic factors (e.g., cytochrome c, SMAC/Diablo) from mitochondria by inducing mitochondrial outer membrane permeabilization (MOMP).
- Proapoptotic, which contain only the BH3 domain (e.g., BID, BIM, PUMA, BAD, NOXA, HRK, BIK, BMF, BNIP3, and NIX) and are activated or induced by cell-death stimuli to promote cell death. This “BH3-only” group can be further subdivided into activators and/or sensitizers (39, 40). BH3-only activators directly and/or indirectly activate effector proteins to induce MOMP (31), and BH3-only sensitizers bind to antiapoptotic proteins to allow activator and effector proteins to drive MOMP (39). BIM has been reported to have the capacity to act as both sensitizer and activator, making it a powerful BH3-only protein actively involved in the response to drug therapy in blood-related malignancies (35, 41–45). BH3-only proteins and their role in apoptosis have been extensively reviewed elsewhere (36, 46).
- Antiapoptotic multidomain proteins (including BCL-2, BCL-X_L_, myeloid cell leukemia-1 [MCL-1], BCL-W, BCL-2-A1, and BCL-B) interact with both the multidomain effector proteins and BH3-only proteins to inhibit MOMP.

Overexpression of MCL-1 is recognized as having a critical role in several hematologic malignancies including diffuse large B-cell lymphoma (47), multiple myeloma (MM) (48), chronic lymphocytic lymphoma (49), and in AML cell survival and treatment resistance (50–52). Further, increased MCL-1 expression is associated with treatment resistance to myelodysplastic syndrome (MDS) (53, 54), which can evolve into AML. Preclinical studies support the potential for inhibition of MCL-1 to attenuate the underlying pathogenesis of AML (48, 50, 51, 55–59), including when used in combination with BCL-2 and/or BCL2L1 (also known as BCL-X_L_) inhibition (60–64). MCL-1 sequesters mitochondrial BAK, thereby preventing homo- or hetero-oligomerization, pore formation, and ultimately, apoptosis. MCL-1 is distinct from other antiapoptotic BCL-2 proteins as it has a very short half-life, ~30 min (56, 65), and therefore requires active transcription and translation to maintain functional levels in the cell (66).

Abundant evidence supports the concept that MCL-1 represents an attractive therapeutic target in AML (59, 67, 68). High MCL-1 expression appears to play a critical role in the survival of leukemic stem cells in patients with AML, and deletion of MCL-1 resulted in the cell death of murine AML cells in mice (50, 69). Further, some patients with AML have shown a ≥2-fold increase of MCL-1 expression at relapse (70). While initially intuitive to inhibit MCL-1 directly, several MCL-1 inhibitors failed in early preclinical development because of their toxicity [the development of small molecule and peptide MCL-1 inhibitors over the past decade has been reviewed in multiple articles (71–74)]. An alternative approach is to indirectly inhibit the antiapoptotic function of MCL-1. Indirect targeting of MCL-1 by inhibition of specific CDK isoforms that regulate transcription, particularly CDK9 or CDK7, has shown promising preclinical and clinical outcomes (8, 29, 30).

## CDK Inhibitors As Cancer Therapeutics

CDKs are a family of serine/threonine kinases discovered in yeast, classically shown to promote cell-cycle transitions via interaction with various fluctuating cyclins (75, 76). A plethora of evidence suggest that the classical cell cycle CDKs, such as CDK1 and CDK2, are targets for treating various malignancies driven by uncontrolled proliferation (77, 78). Interfering with CDK function by targeting CDK regulatory kinases upstream of CDK1/2, such as CHK1 (79) and WEE1 kinase (80), or their combination (81), has also shown proof-of-principle activity in AML (79), and other cancers (82).

The concept of targeting cell-cycle dysregulation as a therapeutic approach has been a major focus of cancer research for many decades. Research and development has been centered around multiple efforts with small molecules directed against CDKs, including for leukemia and MDS (83–85). Several CDK inhibitors have been investigated as cancer therapies (29), with approvals in recent years in solid tumors for palbociclib (86–89), ribociclib (90, 91), and abemaciclib (67, 92–94).

CDKs have other functions beyond direct cell-cycle regulation (Table 1) (66, 76, 114, 124–130). Additionally, CDKs that regulate gene transcription are of increasing interest as potential therapeutic targets in cancer (130–132).

**Table 1:** Functions of CDK isoforms.

**CDK isoform**	**Main function(s) (selected)**
CDK1	Control of M phase of cell cycle; myoblast proliferation (95–97)
CDK2	Control of G1-S phase of cell cycle; myoblast proliferation; Rb/E2F transcription (97–99)
CDK3	NHEJ-mediated DNA damage (100)
CDK4	Control of G1 phase of cell cycle; Rb/E2F transcription (98, 101, 102)
CDK5	Neuronal function (103, 104)
CDK6	Control of G1 phase of cell cycle; Rb/E2F transcription (98, 102, 105)
CDK7	RNA Pol II transcription; CDK-activating kinase (66, 106, 107)
CDK8	RNA Pol II transcription (108–111)
CDK9	RNA Pol II transcription (112–115)
CDK10	Ets2 transcription (116, 117)
CDK11	RNA splicing (118–120)
CDK12	RNA Pol II transcription, RNA splicing (121, 122)
CDK13	RNA Pol II transcription; RNA splicing (123, 124)

*CDK, cyclin-dependent kinase; NHEJ, non-homologous end joining; RNA Pol II, RNA polymerase II*.

### Transcriptional CDKs

As gene transcription has been found to be dysregulated in several cancers, including AML, transcription-associated CDKs are a natural target for cancer therapy. Inhibition of CDK7, CDK8, and CDK9 have been of particular interest in AML (59, 133, 134). These CDKs phosphorylate the carboxyl terminal domain (CTD) of RNA polymerase II (RNA Pol II), to facilitate the production of mature transcripts (128, 129, 135, 136). These transcriptional CDKs have been found to be dysregulated in AML (58, 137).

#### CDK8

CDK8 regulates the Mediator complex, a highly conserved multiprotein complex that functions as a coactivator of transcription (138, 139). Among its functions, the Mediator complex interacts with the pre-initiation complex consisting of RNA Pol II and general transcription factors, such as TFIIH to initiate the process of transcription (140). A CDK8 subcomplex, consisting of CDK8, cyclin C, and Mediator complex subunits Med12, and Med13, prevents re-initiation of transcription after RNA Pol II promoter clearance (i.e., transcription elongation) by binding to the Mediator complex in a manner mutually exclusive with RNA Pol II and independent of CDK8 kinase activity (140). CDK8 kinase activity is reported to both negatively and positively regulate transcription, which may be promoter-specific (141–143). For example, CDK8 phosphorylation of general transcription factors, such as TFIIH or transcription factor Notch, promotes disassembly of the pre-initiation complex to negatively regulate transcription (141, 142), while CDK8 phosphorylation of histone H3 facilitates cooperative histone H3 acetylation that activates transcription (143) by phosphorylating cyclin H (139, 140).

**SEL120-34A** is a novel, potent, and selective CDK8 inhibitor being evaluated preclinically (144). In AML cell lines, CDK8 inhibition with SEL120-34A was effective in cells expressing high levels of STAT5 and STAT1 (144). SEL120-34A also showed *in vivo* activity in mice with xenografted KG-1 and MV4-11 AML tumors with repression of oncogenic MCL-1 in MV4-11 tumors (144).

#### CDK7 and CDK9

**Figure 2:**
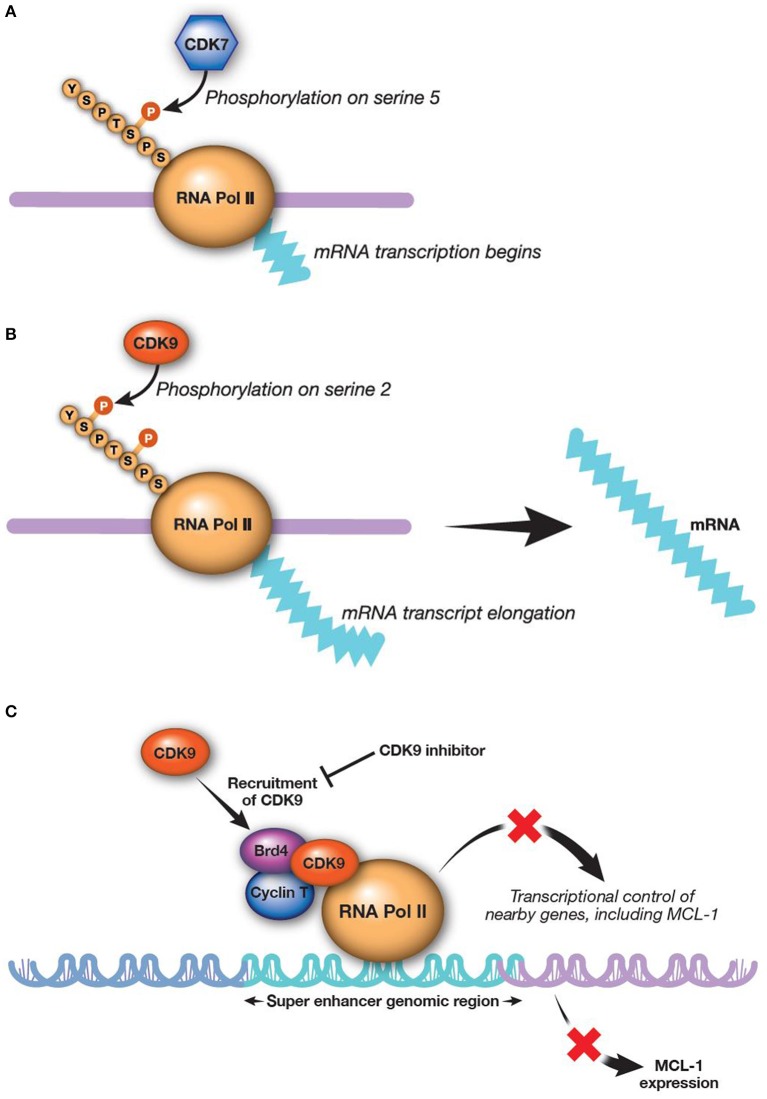
CDK9 and CDK7 work sequentially to produce mature transcripts. CDK9 is the catalytic subunit of the P-TEFb complex, which is essential in generating mature transcripts. **(A)** CDK7 phosphorylates the fifth serine on the carboxyl-terminal domain of RNA Pol II, thereby activating RNA Pol II to begin transcribing RNA^a^. **(B)** CDK9 then phosphorylates the second carboxyl-terminal serine to enable elongation of RNA transcripts (113)^a^. **(C)** Inhibition of CDK9 reduces MCL-1 expression (8). ^a^Reprinted from Morales and Giordano (113), with permission from Taylor & Francis. Brd4, bromodomain-containing protein 4; *CDK*, cyclin-dependent kinase; MCL-1, myeloid cell leukemia-1; *P-TEFb*, positive transcription elongation factor b; RNA Pol II, RNA polymerase II.

RNA transcription is catalyzed by both CDK7 and CDK9 working in sequence (Figures 2A,B). CDK9 is the catalytic subunit of the positive transcription elongation factor b (P-TEFb) complex that, together with a regulatory subunit (cyclins T or K), phosphorylates the CTD of RNA Pol II (145). This process is essential to escape from abortive initiation and facilitate RNA Pol II elongation to generate mature mRNA/transcripts (113, 146–148). The CTD of RNA Pol II is first phosphorylated by CDK7 as part of the transcription factor IIH complex, allowing polymerase to initiate transcription (76, 125, 149). However, the CTD of RNA Pol II must be further phosphorylated by CDK9/P-TEFb for productive elongation of transcription to occur (113, 150). Application of CDK9 and CDK7 inhibitors modulate the activity of all RNA Pol II-regulated genes. There are a few preclinical studies evaluating CDK7 inhibition for AML; however, much more focus has been on inhibition of CDK9, as upregulation of CDK9 is associated with MCL-1 synthesis and treatment-resistance (58, 151).

**THZ1** is a CDK7 inhibitor currently in preclinical study for treatment of AML and peripheral T-cell lymphomas (152, 153). THZ1 prevents Pol II CTD phosphorylation, which disrupts pausing, capping, and elongation, thereby inhibiting transcription (154). In preclinical studies, THZ1 appeared to inhibit transcription factors, such as RUNX1, a key regulator of hematopoiesis that is involved in the common *t*(8, 21) translocation event in AML (152, 154). Further studies are needed to determine appropriate dosing for clinical trials.

**ICEC0942** is an orally bioavailable CDK7 inhibitor also being evaluated in preclinical studies (155). Whereas, THZ1 has a covalent mechanism of action and may interact with other kinases, ICE0942 has a non-covalent mechanism of action, which could possibly limit side effects (154, 155). Oral administration of ICE0942 in mice showed favorable safety with no adverse effects on liver or kidney function (155).

**SNS-032** is an inhibitor of CDK2, CDK7, and CDK9. *In vitro* studies of SNS-032 (BMS-387032) alone or in combination with cytarabine, induced AML cell cytotoxicity through inhibition of CDK2, CDK7, and CDK9 transcription, resulting in the attenuation of RNA Pol II-mediated transcription (156).

**Alvocidib**, previously described as flavopiridol, is a pan-CDK inhibitor structurally related to a natural alkaloid derived from *Dysoxylum binectariferum*, a plant indigenous to India (157). The initial clinical use of alvocidib was based on evidence for the compound as a cell-cycle modulator (157–160). However, further investigation revealed that alvocidib is more potent for inhibiting CDKs directly associated with transcription machinery, most prominently CDK9, to regulate RNA Pol II as described above. Down-regulation of transcription by CDK9 inhibition broadly and preferentially affects proteins with a short half-life, such as MCL-1 (161–163). Consistent with this evidence, alvocidib treatment has been shown to decrease MCL-1 levels in AML and chronic lymphocytic leukemia cells (Figure 2C) (30, 162, 164). Alvocidib induces apoptosis in many tumor cell lines, including those derived from lymphoma, MM, and AML (8). Despite exhibiting its own unique toxicities, alvocidib could speculatively have a more tractable therapeutic index owing to its transient and reversible reduction of MCL-1 via CDK9 inhibition, in contrast to other direct inhibitors of MCL-1. Synergistic therapeutic combinations harnessing lower doses of alvocidib than what may otherwise not be effective as monotherapy, are thus also speculatively attractive, as discussed further in the section Combination Therapy Utilizing CDK9 Inhibition.

**Atuveciclib** (BAY 1143572) is a potent, selective P-TEFb/CDK9 inhibitor (165). Early discovery efforts identified *in vivo* activity in both MOLM-13 and MV4-11 xenograft models of AML with a 14-day continuous oral course of atuveciclib at the maximum tolerated dose. Oral bioavailability was improved over the existing lead compound, and atuveciclib was ultimately selected for phase 1 clinical evaluation on the basis of the totality of its *in vitro* and *in vivo* activity (165). A phase 1 dose-escalation trial of atuveciclib in subjects with advanced leukemia has completed, but results have not yet been reported (NCT02345382).

**Dinaciclib** is a pan-CDK inhibitor that targets CDK9, but for which potency is stronger for other CDKs, namely CDK2 and CDK5 (166). Based on preclinical observations involving mostly AML cell lines, dinaciclib treatments resulted in MCL-1 downregulation (167). *In vitro* and *in vivo* antitumor activity of dinaciclib in mixed lineage leukemia fusion protein AML, regarded as a chemotherapy resistant and poor-prognosis subtype, lends further support to the functionality and therapeutic effects of the anti-CDK9 properties of dinaciclib (168).

**LY2857785** is another pan-CDK inhibitor (including CDK7, CDK8, and CDK9) and also inhibits other kinases (130). As part of efforts to characterize the relative contributions of CDK9 vs. CDK7 inhibition, this study suggests that inhibition of cell proliferation was primarily mediated by CDK9. LY2857785 was shown to confer significant inhibition among all 24 tested hematologic cancer lines, with AML cell lines found to be most sensitive. Similarly, an *in vivo* model (MV4-11 xenograft) showed that tumor growth was significantly inhibited by LY2857785 (130).

**TG02** (169–171) has exhibited an IC_50_ < 10 nM for CDK9 and CDKs 1, 2, 3, and 5 along with potent inhibition, albeit less pronounced relative to CDKs, against other targets that include JAK1, JAK2, and FLT3 (169). Using *ex vivo* expanded AML blast cells (*N* = 16), more potent inhibition of cell proliferation was demonstrated for TG02 relative to SNS-032 and the non-CDK-targeted FLT3 inhibitor sunitinib. CDK9 was identified as the most sensitive target of TG02, effecting RNA Pol II-mediated transcription at lower concentrations than those for SNS-032 while downregulation of MCL-1 was confirmed. Findings from MV4-11 and HL-60 AML models collectively support tumor growth inhibition for TG02-treated animals.

**Voruciclib** is an oral, selective inhibitor of CDK9. Preclinical data has shown that voruciclib in combination with BCL-2 inhibitor venetoclax enhances cell death in AML cell lines (172).

These preclinical studies have shown that targeted CDK9 inhibition results in down-regulation of MCL-1 and subsequent cell death in AML cell lines. Clinical trials have been initiated and further support CDK9 inhibition as a potentially effective treatment for patients with AML.

#### Clinical Development of CDK9 Inhibitors in AML

Alvocidib was the first CDK inhibitor to be tested in a clinical trial (159, 160). As of 2019, ~65 clinical trials had been conducted investigating alvocidib alone or in combination with other antineoplastic drugs. Of these trials, 31 assessed alvocidib in hematologic malignancies, including AML (173). While an overview of clinical trial data is beyond the scope of this review, it is important to note that several clinical trials have been conducted or are in progress with the purpose of determining the optimal use of alvocidib in AML. For example, a randomized phase 2 multicenter study, which compared ACM/FLAM to 7 + 3 in newly diagnosed adult AML patients with intermediate- and adverse-risk cytogenetics, reported the activity of alvocidib when it was used as timed sequential therapy in combination with cytarabine and mitoxantrone (3). The ACM/FLAM regimen led to higher complete response rates compared with patients who received either 7 + 3 alone (46%; *P* = 0.003) or 7 + 3 followed by an additional dose of cytarabine on days 1–5 and daunorubicin on days 1–2 (5 + 2; 57%; *P* = 0.08), and without an increase in toxicity; however, relapse and overall survival rates remained similar (3). Final results of the study data (median follow-up, 1,644 days) confirmed the similar overall survival and higher CR rates in the ACM/FLAM treatment arm compared with the 7 + 3 arm (174). Phase 1 trials of atuveciclib in AML and other acute leukemias (NCT02345382) and TG02 in AML and other advanced hematologic malignancies (NCT01204164) have been completed; however, results have not yet been published. A phase 1 trial of AZD4573 [a CDK9 inhibitor for which preclinical data have been presented (175, 176) but have not yet been published] in relapsed or refractory AML and other hematologic malignancies (NCT03263637) was initiated in October 2017. A phase 1 trial of voruciclib is currently recruiting patients with B-cell malignancies or AML (NCT03547115).

#### Combination Therapy Utilizing CDK9 Inhibition

Experimental evidence suggests that malignant cells, including leukemias, may be dependent on one or more of the multiple antiapoptotic proteins for survival (64, 177, 178). For example, while some cancer cells may depend on MCL-1 for survival, others may depend on BCL-2 or BCL-X_L_ (177). Analysis of BCL-2, BCL-X_L_, and MCL-1 protein levels in primary AML samples (*N* = 577) showed that MCL-1, BCL-2, and BCL-X_L_ exhibited variable protein expression levels, within and across differentiation stages (French-American-British [FAB] classification subgroups), as well as across different cytogenetic and molecular defined subgroups (178). This indicates heterogeneity of antiapoptotic protein expression in AML (64) and supports the concept of dual or triple targeting of BCL-2 family proteins in AML and myeloid malignancies. Conceivably, clonal heterogeneity with regard to antiapoptotic protein expression/dependence may be also observed within a single patient and could promote resistance to therapy. However, for all BCL-2 family members, there is also substantial interpatient variability. Agents that target MCL-1 may have no effect on cells dependent on BCL-2 for survival and vice versa. Thus, combination therapies based on dual-targeting of BCL-2 family members may be attractive strategies for treating AML.

With regard to BCL-2 family inhibition, combination strategies including CDK9 inhibitors aim to overcome MCL-1–dependent drug resistance via transcriptional silencing of MCL-1. CDK9 inhibitors can also overcome intrinsic apoptotic resistance via induction of pro-apoptotic BH3-only proteins, such as BIM, possibly occurring through transcriptional down-regulation of miRNAs that negatively regulate these pro-apoptotic BH3-only proteins (30, 161, 162, 179). Considering these premises, potential synergistic partners for CDK9 inhibitors include small molecules known to inhibit BCL-2 family proteins, such as navitoclax (ABT-263; AbbVie, Inc., North Chicago, IL, USA) and venetoclax (30, 62, 172). Pharmaceutical targeting of BCL-2 alone with venetoclax or BCL-X_L_/BCL-2 combined with navitoclax is active in pre clinical models of AML (177) but only modestly active in the clinic (180). Activity of BCL-2 and/or BCL-2/BCL-XL inhibition can be further enhanced by knocking down MCL-1 (45, 177, 181–183). Combination treatment of voruciclib and venetoclax showed synergistic activity in inducing apoptosis in both venetoclax-sensitive and venetoclax-resistant AML cell lines (172). Concurrent treatment with alvocidib plus venetoclax results in reductions of the half-maximal activity (EC_50_) values for venetoclax in both venetoclax-sensitive and -resistant AML cell lines, and also results in synergy in primary AML samples treated with the combination *ex vivo*, as well as in a mouse xenograft model of AML. The combination of alvocidib and venetoclax was largely dependent on BIM, and thus the combination is mechanistically founded in the intrinsic apoptotic pathway (30). Based on this work, a phase 1b clinical trial to evaluate combined alvocidib and venetoclax in AML was initiated in May 2018 and is in progress (NCT03441555). The combination of dinaciclib and venetoclax is also being actively evaluated in a phase 1b trial in patients with relapsed or refractory AML (NCT03484520), which was initiated in July 2018.

Bromodomain and extra-terminal (BET) family proteins, such as BRD4 interact with cyclin T1 and CDK9 to positively regulate P-TEFb [(183–186); Figure 2C]. Thus, BET inhibitors are emerging as a new, additional therapeutic class for alternatively targeting P-TEFb. Several BET inhibitors, such as CPI-0610 (Constellation Pharmaceuticals, Cambridge, MA, USA) (NCT02158858; AML and MDS), MK-8628 (Merck and Co., Inc., Whitehouse Station, NJ, USA) (NCT02698189; AML, MDS, and diffuse large B-cell lymphoma), and R06870810 (Roche, Basel, Switzerland) (NCT02308761; AML and MDS) are being tested in early clinical trials in AML and other hematologic malignancies, including myelodysplastic syndromes. While BET inhibitors are being investigated as monotherapy, their use in combination with CDK9 inhibitors may show even more promising outcomes for treatment of AML. For example, BET inhibitor BI 894999 has shown activity in AML cell lines, primary patient samples, and xenograft models as a monotherapy and in combination with CDK9 inhibitors LDC000067 and alvocidib (186). Combined BET and CDK9 inhibition results in rapid induction of apoptosis *in vivo* and in cultured cells, perhaps due to a global arrest of transcriptional elongation (186). A phase 1 dose-finding clinical study of BI 894999 monotherapy in solid tumors has been initiated (NCT02516553). It will be interesting to compare the therapeutic window and efficacy between CDK9 and BET inhibitors in AML, as well as the potential for combined BET and CDK9 inhibition.

The combination of a CDK9 inhibitor with an immune checkpoint inhibitor is another intriguing approach for the treatment of AML. Programmed cell death protein 1 receptor (PD-1) is expressed in T-cell subpopulations, and increased PD-1 expression may lead to inhibition of the immune response as AML progresses (187). In murine syngeneic tumor models, use of dinaciclib increased immunogeneic properties of tumor cells, including early expression of type I interferon response genes, and increased expression of PD-1. When dinaciclib was combined with an anti-PD-1 antibody, T-cell and APC activation within the tumor was increased, and antitumor efficacy was improved as compared with either monotherapy (188). The combination of anti-PD-1 (pembrolizumab) and dinaciclib is currently being evaluated in a phase 1 trial in patients with hematologic malignancies (KEYNOTE-155, NCT02684617).

## Proteomic And BH3 Profiling As Putative Biomarker Approaches

In addition to an expanded portfolio of potential treatment options, patients may benefit from proteomic profiling, which may predict response to certain treatments. For example, proteomic profiling of several proteins involved in apoptosis, differentiation, and signal transduction identified clusters that correlated with AML FAB subtypes and cytogenetic defined subgroups. In this large proteomic dataset, differences in BH3-mimetic and BCL-2 protein expression were found in FAB and cytogenetic subgroups, supporting a preferential usage of different molecules within the intrinsic apoptotic pathway in subsets of AML (178). Proteomic profiling may also reveal prognostic subgroups that have not been uncovered through transcriptomic methods. For instance, a study utilizing a reverse-phase protein array (RPPA) found that increased chromosomal region maintenance 1 (CRM1) expression in AML is associated with poor survival. Further, inhibition of CRM1 induces apoptosis in AML cells in a p53-dependent manner (189) (see Figure 1). Protein signatures could therefore predict survival and may be useful in directing therapy, including for BCL-2 targeting drugs and combinations. RPPA methods may also provide greater accuracy into protein expression levels and post-translational modifications in cancer cells, which can lead to novel therapeutics (190). In conjunction with genomic and transcriptomic analysis, proteomics can identify commonalities as well as novel networks in cancer biology (190). However, there are certain challenges in implementing RPPA into clinical practice including a centralized database, high-quality patient samples, and establishment of best practices (191).

BH3 profiling is another approach in predicting response of novel therapeutics in AML cells. BH3 profiling is a functional assay for assessing the apoptotic capacity of a cell. This assay specifically determines how readily cells will undergo apoptosis, i.e., whether the cells are primed for apoptosis, as well as which antiapoptotic BCL-2 family proteins may be most important for survival (192–194). Additionally, BH3 profiling of AML cell lines can indicate possible cooperativity of agents targeting different molecules in the apoptotic process, suggesting that BH3 profiling may function as a potential tool to assess combination therapies in AML (171). Using BH3 profiling, Bhola et al. found that AML and other cancer types are highly heterogeneous in regard to apoptotic priming (192). In primary AML cells and AML xenograft models, BH3 profiling showed a strong correlation between the level of apoptotic priming and the relative sensitivity of the cells to the BCL-2 inhibitor venetoclax (39).

BH3 profiling also has the potential to distinguish clinical responses to azacitidine in AML (64). Vo et al. used BH3 profiling to predict responses to targeted BCL-2 inhibition, as well as to conventional chemotherapy in AML (40). In this study, a subset of patients with low-primed samples displayed a higher risk of relapse. In addition, retrospective studies profiling the BCL-2 family proteins (BCL-2, BCL-X_L_, and MCL-1) identified potential biomarkers of clinical response to venetoclax (180). As the HRK peptide has the highest affinity for BCL-X_L_ and denotes BCL-X_L_ dependency and the BAD peptide denotes BCL-2 and/or BCL-X_L_ dependency, a BAD-HRK mathematical derivation is considered a rough metric for BCL-2 dependency. It was shown that dependence on BCL-X_L_ and MCL-1 correlated with a decrease in response after short-term therapy. Addition of the HRK peptide to BH3 profiling as a *post-hoc* metric predicted that longer-term therapy could be used without development of resistance (180).

NOXA is a pro-apoptotic BH3-only protein that exhibits the greatest affinity for MCL-1 and is thought to specifically bind to and disrupt MCL-1 function *in vivo* (Figure 1) (36, 59). Thus, due to NOXA selectivity for MCL-1, NOXA peptides are thought to be a specific read-out of MCL-1 dependency. Thus, NOXA as a BH3 profiling metric may be an ideal biomarker for CDK9 inhibitors given strong mechanistic links to MCL-1. Mitochondrial profiling in patients with AML treated with alvocidib followed by cytarabine and mitoxantrone (ACM, previously FLAM [ACM/FLAM]) revealed that patients who experienced complete response had higher NOXA priming in bone marrow samples than non-responding patients (164, 195). This rationale has also been used in a study of patients receiving combined vorinostat and gemtuzumab ozogamicin therapy. Responders displayed significantly higher MCL-1 dependence (observed as higher NOXA priming) than non-responders (*P* = 0.027), with a high correlation also seen between MCL-1 dependence and overall survival (*P* = 0.026 per logistic regression) (196). It is unknown which BH3 profiling metrics may be best for predicting response to combination therapies, such as BCL-2 combined with CDK9 inhibition.

It is important to note that, while BH3 profiling has demonstrated clinical utility, a lack of standardized protocols may limit broader clinical applicability. The use of different platforms, as well as the use of either whole-cell or mitochondrial preparations, may lead to different conclusions (197). Difficulty in determining the optimal peptide concentration for profiling highlights further need for standardization (198). Finally, existing BH3 profiling techniques are labor-intensive and require a large volume of starting tissue, which increases error and decreases clinical applicability (199). Despite these limitations, further refinement of BH3 profiling is promising for precision medicine-based treatment.

## Conclusion

Inhibitors of transcriptional CDKs are potential novel therapies for AML. While preclinical studies are currently investigating CDK7 and CDK8 inhibitors, there is abundant preclinical and clinical evidence to support targeting CDK9 whose inhibition downregulates, amongst others, the important anti-apoptotic protein MCL-1. CDK9 inhibitors represent a novel therapeutic class of small molecules for indirectly inhibiting MCL-1. Thus, over-expression of MCL-1 could be a predictive biomarker for treatment response to CDK9 inhibition. BH3 profiling, measuring specific priming for MCL-1 and other BCL-2 family proteins, also has the potential to serve as a biomarker for identifying/selecting patients who could favorably respond to CDK9 inhibition. CDK9 inhibitors have the potential to be combined with other agents in AML, and results are awaited from several completed and ongoing clinical trials.

## Author Contributions

RT and JMB participated in designing the concept of this manuscript, reviewed the literature, and drafted the article.

### Conflict of Interest

RT and JMB report medical writing support for this publication. RT and JMB have published work on alvocidib that is being used by pharmaceutical companies designing clinical trials and did not receive any personal or research compensation for their work.
